# Lecture on Sulphur

**Published:** 1885-04

**Authors:** J. T. Kent

**Affiliations:** St. Louis


### LECTURE ON SULPHUR.

PROF. J. T. KENT, M. D., ST. LOUIS.

CHARACTERISTICS.—The Sulphur patient is a lean, lank, hungry, stoop-shouldered fellow. This patient is content with old rags, thinks they are beautiful; wants to be alone. Sulphur goes as deep into the life as any remedy in the *Materia Medica;* has deeply situated affections. It affects the system slowly and profoundly, being long-acting; leaves marked traces of its action. A prominent symptom is a burning on top of the head and soles of the feet—wants to put feet out of bed. It matters not what the disease is, these symptoms are generally present. Patient gets hungry and faint at 11 o'clock A. M.; there is gnawing at the stomach that compels him to eat. Sulphur diarrhœa drives patient out of bed in the morning. Patient is worse in the morning or after midnight; peculiar fetor of stool, sulphur smell. Although he has not soiled himself, the smell of the stool follows him about. Sulphur aids in absorption and resorption. There is no remedy that will so speedily tone up an old, broken-down constitution as Sulphur. Old deposits in tissues will disappear under its influence; infilteration in the parenchyma of the lungs is taken up and caused to disappear by it. In the treatment of neglected pneumonia, where there are deposits in the lungs, Sulphur causes them to be absorbed.

Fidgetty and morose, wants to be alone, great debility and trembling, talking fatigues. The Sulphur patient says he has flushes of heat and then is cold and has slight sweat. Unsteady gait, tremor, walks like an old man, uncertain in his movements. In epilepsy patient falls to the left side. Worse in the open air, better in warm room. Standing is the most disagreeable position the patient can assume. The vertigo of Sulphur worse in open air, while Puls. is better in open air. In Sulph. chest affections are *better* in *open air*, the *brain* affection better in a warm room.

A baby will need Sulphur where its mother has been dirty. Whenever indicated in the nursing mother give it to the child. Sulphur antidotes Aloes.

MENTAL.—Weak memory, softening of the brain. Despondent, forgetful, inability to concentrate his mind. Foolish happiness and pride. Disgust, even to nausea, of any effluvia, even of his own body; disgust for any smell that may be offensive. Over-zealous people who have exhausted mind and body in pious duty. (*Aurum, Puls., Psorinum, Ars.* have this morbid religiosity.) (Lach. has religious melancholy.) He is feverish; very often dizzy, which comes on from determination of blood to the head; head hot in occiput to touch (Graph.).

HEAD.—Rush of blood to the head, roaring in the ears, *worse* when *stopping, better sitting in warm place* (sometimes *aggravated* by *heat*, so that he must have doors and windows open). *Generally* Sulphur patient is *cold*, while his *feet* are *burning;* irregular, faulty circulation. Heaviness and fullness in forehead when rising to a sitting posture. *Worse after sleep*, wants to sleep *late in the morning;* jerks in sleep; wakeful about *two or three o'clock in the morning*, then sleeps until *eight or nine; awakening unrefreshed.* Dizziness whenever patient crosses water, sometimes nausea, hence a supposed remedy in sea-sickness. Aggravated from *exercise* and *drinking milk* (like Merc.), worse from sweat. Sulphur has almost every kind of pain in the head. They are attended with throbbing and sickness of stomach. The pains are *relieved* by *warmth, aggravated* by *coldness.* Most of them *come on during rest* and *pass off during motion; standing* is the most disagreeable posture. Sulphur affects the *right side* more than the *left.* Periodicity every eight days. Pains *aggravated* at *night* and when *weather changes.* Pains in head and stomach come on while walking in open air. Yellow green spots, filled with vermin, upon the scalp (also Kali-sulph). (Tongue red, slick and shining—Kali-bich.). Voluptuous itching is *relieved* by *scratching;* the *scratching* is *very agreeable.* Little hard bumps often appear on the skin on scratching. Sulphur patient dreads washing. Inflammation of eyes made *worse* by *washing; water irritates the skin.* (Puls. patient *likes* to be *washed.*) Humid, offensive ulcers, with thick, yellow pus, itching, tingling, burning, and scalding. (Dulc., Rhus, and Graphites.) Sensitiveness of the vertex, worse in the evening; smarting and burning after scratching; sensation as if a band was around the head (like Merc.); Lach. and Nitric acid have sensitiveness of vertex. (Schoolgirl gets headache from weight of hat—Nitric acid.) (If headache comes on every two weeks, Ars., but don't repeat it, else you will not cure your case.) (Sulphur, Silicia, and Sanguinaria have headache every eight days.) In Sulphur the fontanells close too late (for which Calc. is usually given).

EYE.—Ocular illusions and stitches in the eye, *worse* in *hot weather;* perspiration round about the lids produces a smarting and burning; retinitis. (Glonoine will cure a case coming on from too bright a light and intense heat.) Pustular inflammation of cornea, ulceration of the lids, margin (also Apis, Merc., and Euphrasia).

Ear.—Purulent otorrhœa; the discharge is offensive; it may be thick or watery; it is the result of a chronic trouble; catarrhal discharge from the ear; *worse* from washing; patient is *filthy*. Sulphur leads the list for catarrhal symptoms in general. When *well-selected remedies fail to act* it brings out the symptoms, develops the case, sometimes brings eruptions out upon the surface. When the disease is getting well it cures from *above down*—*surface manifestations* are always *favorable*. Disease also recovers from *within outward*.

Nose.—Great dryness in the nose and formation of dry scabs; a profuse discharge of white globules of mucus; *early* in catarrhal state is *fluent*, *later* becomes *dry;* profuse catarrhal discharge of *burning water*. It has chronic stoppage of *one nostril*. Mucous membranes have become hypertrophied. These catarrhal symptoms are accompanied by *hot feet* and *heat* on *top* of the *head*, lean, lank, hungry, stoop-shouldered. If caused from a cold he has taken, give Sulphur high (a high potency of Sulphur acts from six weeks to two months). Freckles and black pores on nose. (Bovista patient has nose always filling up with black scabs.) Swelling and inflammation of the nose. (Strawberry nose—Lach.) Sulphur produces a cachectic, care-worn, crabbed, sickly countenance, circumscribed redness of cheeks, hectic fever. (In last stages of consumption, with high fever, *where Sulphur will no longer act*, intense headache, with burning in the skin, etc.; sensation as if stuffed up; can't expectorate. Give one dose of *Phosphorus high*, and *don't repeat it;* aggravation may be a profuse diarrhœa.)

Mouth.—Lips brown, cracked, dry; herpes around the mouth (Natrum mur.). After Rhus and Bell. have been indicated and have exhausted themselves, Sulphur comes in to finish the case. (In bleeding gums Merc. must be thought of before Sulphur.) Tongue red at the tip and bleeding, mucous membrane red, mucous membrane of lips red and angry. Ptyalism from abuse of Mercury. *Saliva profuse and of a nauseous taste*, which is very characteristic. Bad smell from the mouth, mostly after eating. Thrush (Sulp. acid will cure the most cases of thrush). Sensation of a lump in the throat (if it comes up and patient must swallow, when it descends, but returns at once—*Lach.*). (If lump comes up from *low down*, attended with *sighing* [globus hystericus]—Ignatia.) Tongue coated early in the morning passes off during the day. Great burning and dryness, first right then left side of throat. Where Lyc. does not finish case, Sulphur may do good.

GASTRIC.—Great craving for food in big-bellied, dirty children; *drinks much* and *eats little;* violent thirst for beer; longing for brandy (*Nux vom.*); desire for sweet things. (Where mother has eaten much candy and child gets diarrhœa—Argent-nit. also Sulphur.) (Argent-nit. has a stool very like *Merc.*, grass green, straining and crying.) Sulphur predisposes to a weakness of whole system; weak stomach and a variety of dyspeptic symptoms; sore stomach, vomiting two or three hours after eating, nausea in the morning after eating. Eructations generally empty and tasting of the food. Important in morning nausea during pregnancy (*Ars.*, *Puls.*, and *Sepia*). Great desire for food, which gives place to disgust at the sight of food. Vomiting at 5.45 P. M. regularly every day in an *enciente* female. Marked weakness about 11 A. M. in stomach, empty, gone, faint feeling—this is one of the first symptoms of Sulphur. Pressure at the stomach after eating—a great many remedies have this—if taking food *relieves, Sulphur.* If eating *does not relieve*, and patient is hungry at *any time, Sepia.* (Ignatia and Hydrastis also have this capricious appetite, but it is in *nervous* people. In Sepia it is brought on by disturbances in the *uterus*, "I have an empty sensation in my stomach;" Ignatia in connection with *sighing.*)

Sulphur is a great liver remedy. Swelling and hardness—where this has been cured, Sulph. symptoms have been present. Stitches in spleen, *worse on deep inspiration.* (Hypertrophy of the liver has been cured by *Kalmia*, in connection with heart-disease.) Intestines feel as though strung in knots, *worse on bending forward.* Big-bellied, with emaciated limbs, in weak children (Baryta carb., and Calc.).

STOOL.—Sulphur has a very wide range of action on the bowels. Diarrhœa coming on in the morning; sphincters are relaxed, and they let loose on him. *Driven out of bed in the morning;* stool painless, pappy, may be involuntary. He is afraid to pass flatus. (Lack of confidence in the sphincters is also characteristic of Oleander and Aloes.) Stools excoriate the anus. Painfulness of stool and urine to the parts over which they pass. Constipation—round, black, hard balls is Opium; if Sulphur characteristics are present Opium will not cure. Sulphur is a leading remedy in hemorrhoids (Nux vom.). The patient has enlarged veins, sometimes suppuration of these tumors. Often the suppuration ceases, and the patient gets a cough; when the flow commences again the cough passes off (Berberis also). Anus sore, with itching, in old men, as soon as they get in bed. Dry, cracking of skin.

URINE.—Frequent micturition at night; large quantities of colorless urine after hysteria. If child kicks cover off in sleep, and has incontinence of urine at night. (Child wants covers on —Calc.) Sulphur patient may feel cold to the touch, yet be burning internally. (Calc. child wants to lie quiet and well-covered; is cold.) Urine fetid, greasy pellicles on surface, like coal oil on water; variegated in color. (Where there is sand in urine with pellicle—*Phos.*) Mucous discharges from the urethra. When patient gets gonorrhœa, if he has formerly had Sulphur characteristics, Sulphur will cure him. Sulphur and Sepia produce nearly all the states, stages, and appearances of old debauchees who are impotent. Coldness of penis and impotency.

Sulphur is a great remedy for enlarged prostate gland—will cure fifty out of every one hundred cases without discrimination.

MENSES too late and too short. (*Puls.* patient wants to get out of doors; Sulphur also. *Puls.* has *burning on top of feet and ankles.* Puls. has sour stomach; bloated.) Amenorrhœa, blood thick, acrid, sour-smelling mucus. The discharges are *burning;* stool also.

Leucorrhœa, *burning*, corrosive. Burning in the vagina, with dryness; is scarcely able to keep still. Sore feeling in vagina during coition. (Schussler's Ferrum-phos., Sepia, Platina.) Troublesome itching of the vulva and surrounding parts—itching, burning, pricking, till they nearly go frantic (Apis). (*Apis—kicks the clothes off; wants doors and windows open;* would like to be dashed with *cold water; cold relieves.* Labor-like pains over the symphysis.) (Throbbing behind the pubes—Æsculus hip.) (Delay of the first menses—Puls. and Calc-phos. cover the symptoms of most cases.) Females with weak genitals. If there be any predisposition to consumption or scrofula, Sulphur will do the baby good. Women who have abscesses of the breast. (Bell. and Phytolacca will prevent the formation of abscess in the udder.) If a case has been spoiled by malpractice, and Sulphur symptoms are present, give it. After you have vaccinated a child, give it Sulphur high. (Thuja, Silicea, or Calc. may be called for.)

CHEST, LARYNX.—Talking fatigues and excites pain. Shooting pain through left chest to back. (Ars.—pains in the supra-clavicular space.) *Worse lying on back.* (Phos. has pains in *left* chest; relieved by lying on *right* side.) Catarrh with fluent coryza. (Rawness in the trachea, Carbo veg., Stannum, and Phos.) Nightly suffocating fits; wants doors and windows open. (Secale patient wants *doors* and *windows* open, but his skin is cold and blue.) Difficult breathing. Great throbbing of the heart, which causes external manifestation. Rattling in the chest, *worse after* expectoration. Sulphur is allied to Kali-sulp., the cough is dry, expectoration difficult, and yet there is *rattling* as if a *great amount*. Kali-sulp. has yellow expectoration, while Sulphur has white. Patient sometimes spits up considerable blood, mucus, and pus (Silicea also). (Every cold patient takes settles in the chest—Phos.) Chronic catarrh of the chest. In the so-called " quick consumption " Sulphur is the most valuable remedy. The patient has had a winter cough for ten years, and finally a rattling comes on, but patient does not expectorate ; it is a mucous consumption. Miliary tubercle. No dullness on percussion, as with tubercle, because it is simply mucus. There are night-sweats and hectic fever, high temperature, and quick pulse.

PNEUMONIA.—Not for first stage. It may be Bryonia, Aconite, or Veratrum viride. The acute stage has passed off and there is filling up (pneumonic-hepatization), dullness. This stage is preceded by pain and excitement, but when hepatization comes on pains leave, and patient *thinks* he is better, but if he is examined closely you will discover that he is not. (Sulph., Phos., and Lyc.) Cough excited by tickling in larynx as from down, evening and night ; sputa of a dark, greenish purulent, or milky white watery ; it is of a sourish, offensive taste, like discharge of an old catarrh ; in maltreated pneumonia or in some catarrhal conditions of the chest we have this symptom frequently ; burning in the chest rising to the face. In exudation after pneumonia, don't forget this *remedy*.

HEART.—It is the remedy to begin with in one-sided cases, and afterward select the right remedy. (Spongia and Lach. are prominent heart remedies, and follow Sulph. well.) (Lach. has more heart symptoms than any other remedy in the *Materia Medica*).

BACK AND EXTREMITIES.—Stiffness in the neck or back. Sulphur may be the first remedy to administer if you have these characteristics. (Calcarea, Puls., Silicea, and Sulphur will cure the majority of cases of spinal curvature.) Rheumatic pains in the shoulders. A prominent symptom of Sulphur is sweating in the axilla, smelling like garlic. It is not a great rheumatic remedy, like Rhus and Bryonia, but if you have Sulphur symptoms present you cannot cure until you have administered a dose of Sulphur first. Sulphur very often selects the knee-joint as its seat of action, hence you will find an enlarged joint, thickening and hypertrophy, great pain and swelling. With its characteristics Sulphur will begin the cure. Burning of soles of feet, wants them uncovered, also soles cold and sweating. In hip-joint disease, with characteristic pus and burning of extremities. Chilblains thick and red. Limbs go to sleep while sitting on buttocks. Sulphur produces a stagnation of the veins. High livers sometimes have gout, chalky deposits in joints. Sometimes feet swell. Dropsical swelling of legs up to knees. There are only two remedies that have "relief in rheumatism from putting feet in cold water"—Puls. and Ledum. Pain aggravated from covering with feathers. (A rheumatism that is deep seated in the tendons *aggravated* by *warmth* of *bed* will call for Merc.) In rheumatism the skin affection is soothed by something cool. Sticks feet out from under bedclothes until cool, then puts them under again. (Ars. patient is made better by *warm* applications in rheumatism, while pains in the head are made better by *cold.*) *Cold relieved* the headache. Sulphur may be indicated in "*milk leg*" (Calc. and Sepia).

SLEEP.—The sleep is characteristic—sleepy all day and awake all night. (Phos.) Sulphur patient is sleepy the fore part of the night, and from midnight until two or three o'clock is wakeful; rouses up as if frightened; his feet burn, feels as if he has too much clothing on; goes to sleep about daylight, sleeps until late in the morning, when he awakes feeling worse than before; stupid; dull. (Nux v. and Puls. have a similar state.) The Nux v. patient wakes up about 3 A. M. and thinks of his business affairs. The Nux v. patient is more quiet (Sulphur is restless).

SKIN.—The skin itching worse from warmth. Sometimes Sulphur is indicated in advanced stages of scarlatina. Chill, fever, sweat in the advanced stage. Skin easily excoriated (if after this there is an exudation of a glutinous character—Graphites). Soreness of the folds of the skin, in abscess, furnuculus, glandular swellings, indurated or suppurated. Hands chapped every time water is applied; burning is a prominent symptom. Sulphur, Calcarea, Lyc. follow each other well.

